# Remineralization potential of dentifrices with calcium sodium phosphosilicate and functionalized tri‐calcium phosphate in the deeper incipient carious lesions: An in vitro study

**DOI:** 10.1002/cre2.876

**Published:** 2024-03-20

**Authors:** Shafiq Aziz, Carolina Loch, Kai Chun Li, Robert Anthonappa, Alison Meldrum, Manikandan Ekambaram

**Affiliations:** ^1^ Sir John Walsh Research Institute, Faculty of Dentistry University of Otago Dunedin New Zealand; ^2^ Paediatric Dentistry, UWA Dental School The University of Western Australia Perth Australia

**Keywords:** dentifrices, enamel caries, fluorides, tricalcium phosphate

## Abstract

**Objectives:**

This study evaluated the remineralization potential of calcium sodium phosphosilicate and functionalized tri‐calcium phosphate (f‐TCP) dentifrices in deeper incipient carious lesions (ICLs).

**Materials and Methods:**

Artificial ICLs were created by placing premolars into demineralizing solutions. Teeth were randomly assigned into four groups: calcium sodium phosphosilicate (Group 1), f‐TCP (Group 2), 1450 ppm fluoride (Group 3), and distilled water (Group 4), which were subjected to 10‐day pH cycling. Mineral density (MD) was assessed using microcomputed tomography (Micro‐CT), while hardness (H) and elastic modulus (EM) were assessed using nanomechanical testing.

**Results:**

MD % gain was higher in Groups 1–3 than in Group 4. In addition, Groups 1 and 2 exhibited significantly higher MD % gain than Group 3. Also, Groups 1–3 showed significantly higher EM and H values than Group 4 in the outer enamel area; yet, Groups 1 and 2 displayed significantly higher EM and H values than Groups 3 and 4 in the inner enamel.

**Conclusions:**

The MD, EM, and H of ICLs significantly increased with the addition of calcium sodium phosphosilicate or f‐TCP to fluoridated dentifrices compared to standard fluoride dentifrices. The added active ingredients remineralized the deeper parts of the ICLs, while remineralization at the lesion surface was similar between tested dentifrices.

## 1 INTRODUCTION

Dental caries is a global problem. The World Health Organization (WHO) lists caries as the fourth most expensive chronic disease (Petersen, 2008). In 2010, untreated caries in permanent teeth was the most prevalent health condition globally, affecting 2.4 billion people, while untreated caries in primary teeth affected 621 million children worldwide (Frencken et al., 2017; Kassebaum et al., 2015). Globally, for every 100 individuals, an average of 15 and 27 new cases of dental caries develop each year in primary and permanent teeth, respectively (Frencken et al., 2017; Kassebaum et al., 2015).

The first stage of enamel caries, incipient carious lesion (ICL), comprises an intact enamel surface with reduced mineral content in the subsurface. Treatment and stabilization of an ICL require noninvasive methods such as remineralization with suitable agents or sealing the ICL using restorative materials (Paris & Meyer‐Lueckel, 2010; Shen et al., 2011). Substantial evidence supports the beneficial effects of fluoride dentifrices in preventing and remineralizing dental caries (Amaechi et al., 2012; Marinho et al., 2003; Mensinkai et al., 2012; Walsh et al., 2019). Fluoride dentifrices with additional active ingredients such as calcium sodium phosphosilicate and functionalized‐tricalcium phosphate (f‐TCP) have been introduced in the market, showing promise in the remineralization of ICLs (Parkinson et al., 2017).

Calcium sodium phosphosilicate (Novamin®) is a biomaterial used to manage dentine hypersensitivity by physically occluding the dentinal tubules. It can also be used to enhance the remineralization of tooth surfaces (Burwell et al., 2009). Novamin® contains sodium‐calcium‐phosphate particles that release sodium ions when activated in an aqueous environment. The calcium and phosphate ions form a calcium‐phosphate layer upon precipitation, creating a carbonate‐enriched hydroxycarbonate apatite (HCA) layer. The HCA layer structure and chemical composition are similar to natural tooth minerals, leading to the remineralization of ICLs (8).

Functionalized tri‐calcium phosphate (f‐TCP) is formed when ß‐tricalcium phosphate (ß‐TCP) reacts with organic and/or inorganic components, such as carboxylic acids and surfactants (Karlinsey & Mackey, 2009; Karlinsey et al., 2010). f‐TCP is incorporated with sodium fluoride and marketed as “Clinpro®” (3 M ESPE). Clinpro®'s f‐TCP technology can deliver targeted and sustained fluoride, calcium, and phosphate release. In addition, the manufacturers of Clinpro® claim that it possesses a fumaric acid barrier, which is formed from ball milling ß‐TCP with sodium lauryl sulfate (Karlinsey et al., 2010). This barrier helps prevent undesirable reactions among the individual ions and aids in the coexistence of calcium and fluoride ions during the products' storage. During use, the barrier is broken in contact with saliva, releasing ions for effective tooth remineralization with f‐TCP and fluoride, which is more acid‐resistant (Seyedlar et al., 2019).

Microcomputed tomography (Micro‐CT) has become a standard technique for studying in vitro enamel lesion development (Elliott et al., 2005) and the mineral content of enamel (Dowker et al., 2004; Wong et al., 2004). The main benefits of Micro‐CT are its nondestructive characteristics, delivery of high‐quality analytical data, and internal visualization in three dimensions. There are several ways of quantifying the mineral density (MD) of enamel using Micro‐CT primarily based on the attenuation of X‐rays transmitting through the object and the consequent grey level of the reconstructed image (Dowker et al., 2004; Wong et al., 2004). Nanoindentation mechanical testing measures the hardness (H) and elastic modulus (EM) across a specimen. The process involves an indenter tip driven into and away from a sample; the force and displacement are measured using sensors, and the mechanical properties are then calculated (Oliver & Pharr, 1992). Small indentations formed during nanoindentation mechanical testing mean lesser material pile‐up and a reduction in other strain‐dependent effects compared to microhardness testing. Having smaller indentations also allows for multiple rows of indentations across the lesion. This measurement is essential when analyzing DE lesions, as surface hardness alone does not accurately describe the whole lesion and the depth into which tested agents may penetrate. The substrate to be tested needs to be sectioned and finely polished, as surface imperfections and scratches will result in inaccuracies during nanoindentation testing (Gadelrab & Chiesa, 2013). Although used in recent studies (Dowker et al., 2004; Elliott et al., 2005), surface microhardness does not accurately represent the mechanical properties of the body of the enamel lesion. H and EM derived from nanoindentation across the body of the lesion could reflect the actual mechanical properties of the ICLs (Wong et al., 2004).

Previous studies showed remineralization of enamel with dentifrices containing calcium sodium phosphosilicate or f‐TCP (Buckshey et al., 2019; Matsuyoshi et al., 2017). However, it is not known whether these dentifrices provide any enhanced remineralization of the ICLs compared to standard fluoride dentifrice, particularly in the inner layers of the ICLs. This study assessed whether fluoridated dentifrices with calcium sodium phosphosilicate and f‐TCP resulted in improved remineralization of deeper ICLs when compared to standard fluoride dentifrice, measured via Micro‐CT and nanoindentation testing. The two null hypotheses are as follows: (1) there was no difference in MD of ICLs treated with fluoridated dentifrices with added active ingredients compared to standard fluoridated dentifrice; (2) there was no difference in nanomechanical properties of ICLs treated with fluoridated dentifrices with added active ingredients compared to standard fluoridated dentifrice.

## 2 MATERIALS AND METHODS

### 2.1 Specimen preparation

**Figure 1:**
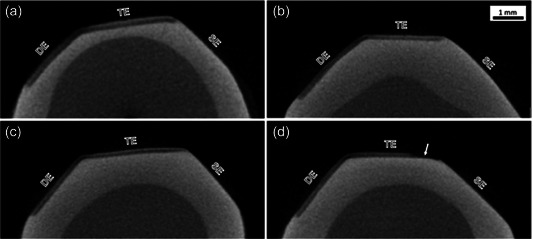
Micro‐CT images of representative specimens showing demineralized enamel (DE), treated enamel (TE), and sound enamel (SE). Remineralization of the lesion is evident in Group 1, SRP (a) and Group 2, CTC (b). Slight remineralization of the lesion is evident in Group 3, CCP (c). No remineralization and partial erosion of lesion (arrow) are seen in Group 4, DI (d).

Ethical approval was obtained from the University of Otago Human Ethics Committee (Health); Reference number [H19/047]. After power calculation, a sample size of 32 specimens (*n* = 8 per group) was used. This estimate was based on the calculated sample size required to achieve a power (π) of 0.80 at the significance level of α = 0.05 using G*POWER 3.1.9.4 software, where the conventional value of β = 0.20 as a standard of adequacy. A conventional value *ƒ* = 0.25 for medium effect size was used. Human premolar teeth extracted for orthodontic reasons were collected from patients after signed consent. Only sound teeth with no caries, developmental enamel defects, anomalies, or restorations were included in the study. Extracted teeth were cleaned with a scalpel, slow‐speed handpiece, and rotary brush to remove plaque, blood, and other contaminants and were subsequently stored in thymol solution at 4°C. Teeth were decoronated, and the crowns were later sectioned vertically into two halves (buccal and lingual/palatal) using a diamond wafered blade on a high‐speed saw under water irrigation (Struers). The outer prismless enamel layer was removed with 1200 grit silicon carbide paper under running water on an automated polisher (Tegra‐Pol‐21, Struers). Three zones were demarcated: sound enamel, demineralized enamel (DE), and treated enamel; Figure 1. All the crown surfaces, except for a 4 × 4 mm^2^ window encompassing two of the three prepared planes, were covered with two layers of acid‐resistant nail varnish (Revlon).

### 2.2 De/remineralizing solution (RS) preparation

The demineralizing (DS) and RS were prepared following the saturation concentration of hydroxyapatite minerals present in saliva under normal conditions, as used in previous studies (Gopalakrishnan et al., 2017; Kumar et al., 2008). The DS consisted of 2.2 mM CaCl_2_, 2.2 mM KH_2_PO_4,_ and 0.05 M acetic acid adjusted with 5 M KOH to pH 4.4. The RS consisted of 1.5 mM CaCl_2_, 0.9 mM NaH_2_PO_4,_ and 0.15 M KCI adjusted with 5 M KOH to pH 7.0. The pH adjustment was assessed using a pH electrode (Jenco Model 6010 M, Jenco Instruments) calibrated to two solutions of known pH: 4 and 7.

### 2.3 DE lesion formation

The prepared enamel specimens were immersed in a DS solution (20 mL/specimen) on an orbital shaker (Labnet) at 80 rpm. The immersed samples were kept at 37°C for 96 h in an incubator to facilitate the formation of DE lesions. The solution consisted of 8.75 ml 85% lactic acid, 0.16 mM Ca(H_2_PO_4_)_2_ diluted to 1 L distilled (DI) water and adjusted with 1 M KOH to pH 4.4 (Bijle et al., 2018; Bolis et al., 2015). In preparing all test solutions, appropriate analytical‐grade chemicals were thoroughly mixed in distilled water using a magnetic bar and stirrer. The solution was changed every 24 h. After DE lesion formation, the specimens were removed from the solution, thoroughly rinsed with DI water, and dried on fibreless laboratory napkins (Kimwipes™ Ex‐L, Kimberly‐Clark Professional). For each specimen, one of the two exposed planes with DE lesions was covered with acid‐resistant nail varnish (Revlon) to act as the demineralized control for that specimen.

### 2.4 Test groups

The prepared enamel specimens were randomly allocated into the following groups (8 specimens/group), as follows: Group 1: Sensodyne repair and protect® containing 1426ppm fluoride and calcium sodium phosphosilicate (Novamin®); Group 2: Clinpro tooth crème® containing 950ppm fluoride and functionalized‐tricalcium phosphate (f‐TCP); Group 3 (positive control): Colgate® Cavity Protection containing 1450ppm fluoride; Group 4 (negative control): Distilled water.

### 2.5 Preparation of treatment agents

Dentifrice slurry for groups 1, 2, and 3 was prepared in a standard 1:3 ratio of dentifrice to DI water (15 g of dentifrice to 45 mL of DI water). The dentifrice and DI water underwent thorough mixing and mechanical agitation for 5 min using a vortex mixer (Super Mixer, Lab Line Instruments Inc.) at room temperature. Next, the thoroughly mixed solutions were centrifuged at 4000 rpm (Beckman, Avanti J‐251, California, USA) for 20 min at 25°C. Subsequently, the sediments were discarded, and the supernatant was used during pH cycling (Kumar et al., 2008).

### 2.6 pH cycling

**Figure 2:**
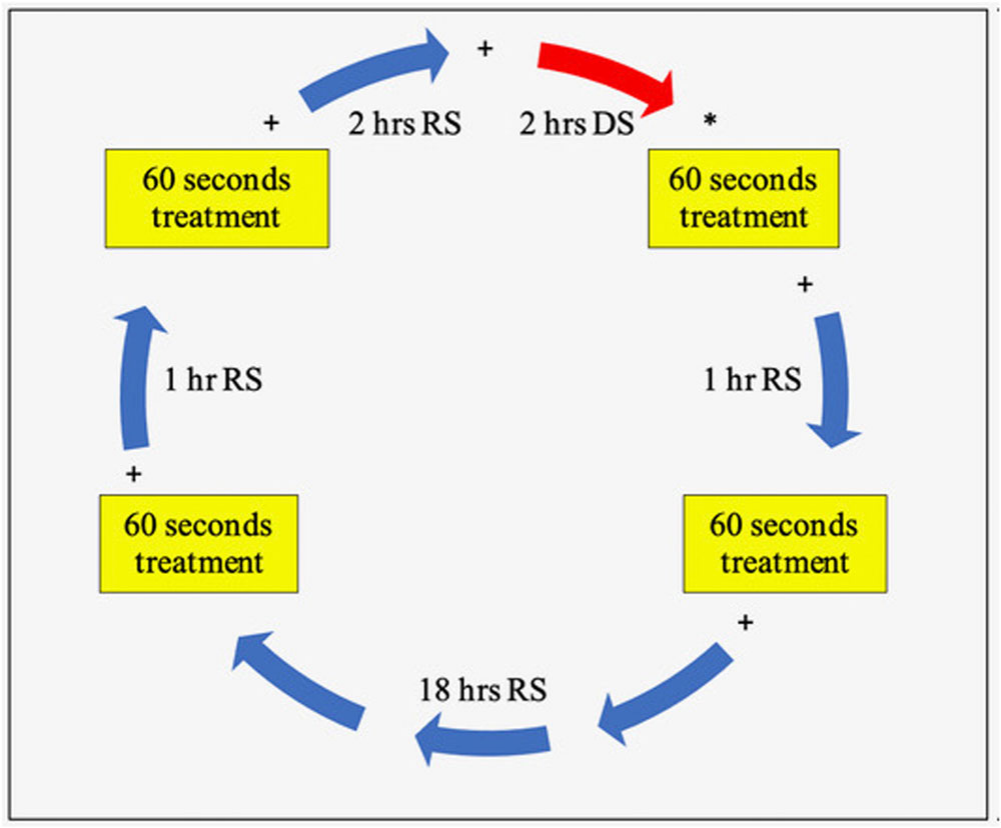
24 h pH cycling model + solution change. *Start of the cycle.

The prepared enamel specimens were subjected to a pH cycling model for 10 days (Cheng et al., 2015). The daily pH cycling model involved 2 h of demineralization with four treatment phases with test solutions for 60 s each. During the remaining time, the specimens were immersed in RS. The four treatment phases involved two treatments before and two after the 2‐h demineralization phase. The first treatment was initiated 3 h before the demineralization phase, followed by the second treatment phase at a 1‐h interval. The third treatment began after 2 h of the demineralization phase, following which the final treatment was accomplished 1‐h post‐third treatment phase. DS (20 mL/specimen) was prepared fresh daily before the DS phase of the pH cycle, while RS (10 mL/specimen/phase) was prepared twice daily and changed after each treatment phase. The pH of the solutions was confirmed at the start and end of each solution change. Specimens were treated with 5 mL of treatment solution at the individual treatment phase. Samples were thoroughly washed with DI water for each solution change and dried on dry fiberless laboratory napkins (Kimwipes™ Ex‐L, Kimberly‐Clark Professional). The entire pH cycling model was performed on a continuously operated orbital shaker (Labnet) at 80 rpm (Bijle et al., 2018). Figure 2 summarizes the pH cycling model.

### 2.7 Micro‐CT MD assessment

Specimens' MD was evaluated using a Micro‐CT scanner (Skyscan 1172 X‐Ray Microtomograph) with constant scanning parameters. Individual specimens and reference phantoms were mounted on a 3D printed stand secured to the computer‐controlled turntable so that the X‐ray beam was perpendicular to the surface of interest. The scanning parameters were 80 kV, 100 µA, Al + Cu filter, 17.3 µm camera resolution, 360° rotation, and 0.3° steps (Bijle et al., 2018). MD calibration was done by scanning reference phantoms comprising three hydroxyapatite disks with known concentrations (0.145, 0.596, and 1.249 g/cm^3^) and corresponding MD (1.165, 1.469, and 1.747 g/cm^3^, respectively) (Schwass et al., 2009). The 3D scanned images were reconstructed using NRecon v. 1.7.0.4 (Skyšcan). The reconstruction settings were as follows: smoothing: 1, misalignment compensation: 0.5, ring artifact correction: 20, beam hardening correction: 30%, and cs rotation: 0°. The processed images with a trans‐axial view were selected for MD assessment. MD was analyzed using CTAn v.1.16.1.0+ (SkyScan) at three different zones per slice (sound enamel, treated enamel, and DE). Zones were labeled as SE (baseline, Sound Enamel), DE (Demineralised Enamel), and TE (Treated Enamel). Three round regions of interest (ROI) of 8 pixels in diameter were used for MD evaluation at randomly selected sites within each zone. MD was evaluated every 20 slices, obtaining 22 slices per specimen. A total of 66 ROI per zone were evaluated per specimen. A mean MD value was calculated to obtain MD at SE, DE, and TE. The mineral gain and percent remineralization were computed using the following equations:

MineralGain=MDdifferencebetweenTEandDE

PercentRemineralisation=MineralGain/(MDdifferencebetweenSEandDE)~100.

### 2.8 Mechanical characterization via nanoindentation

After the MD assessment, 12 specimens (three specimens per group) were selected and cross‐sectioned for nanoindentation testing to measure hardness and EMs values across the lesions. Each sample was embedded in a cold‐curing epoxy resin (Epofix, Struers). Upon curing, specimens were cross‐sectioned using a diamond wafered blade on a high‐speed saw (Diamond Cut‐off Wheel M0D13, Struers). Cut surfaces were polished with silicon carbide paper 1200 grit (Struers) under water irrigation. Final polishing was achieved with 1 µm diamond polishing paste (DP‐Paste M, Struers) on a polishing felt (ASFL Magnetic Cloth, Kemet). Specimens were ultrasonically cleaned between each polishing cycle with distilled water and after final polishing with 100% ethanol for 3 min each (Shahmoradi et al., 2017). Nanoindentation testing was conducted using an Ultra Micro Indentation System (UMIS‐2000, CSIRO), equipped with a three‐sided Berkovich indenter tip. Specimens were nanoindented using a maximum load of 50 mN. Two rows of 22 indents, 60 µm apart, were positioned across the lesion in each sample. The two rows were labeled as the outer layer at 30 µm depth from the surface and the inner layer at 90 µm. UMIS software (IBIS2000, Semi‐Labs) was used for the calculation of the EM and hardness (H) following the Oliver and Pharr method (Oliver & Pharr, 1992).

### 2.9 Statistical analysis

Statistical analysis was performed with SPSS v. 27 (IBM SPSS® Statistics Inc.). Values were checked for normal distribution. Then, one‐way analysis of variance (ANOVA) analyzed statistical differences for MD, and two‐way ANOVA analyzed statistical differences for mechanical properties' values among the test groups with the post‐hoc Games‐Howell and Bonferroni's correction, respectively. The statistical significance level was set at .05.

## 3 RESULTS

### 3.1 Micro‐CT MD assessment

The mean MD of SE ranged from 2.87 (±0.14) g/cm^3^ to 2.95 (±0.06) g/cm^3^, and 1.89 (±0.08) g/cm^3^ to 1.97 (±0.08) g/cm^3^ in DE (Table 1). There was no statistical difference between mean MD values among test groups. The mean MD of TE ranged from 1.99 (±0.08) g/cm^3^ to 2.34 (±0.06) g/cm^3^ (Table 2). When considering the % Mineral Gain, dentifrice test groups with calcium sodium phosphosilicate (Group 1) and f‐TCP (Group 2) and the positive control (fluoridated dentifrice, Group 3) had significantly higher % Mineral Gain than the negative control (DW, Group 4) (*p* < .01). Although there was no significant difference between the % Mineral Gain of Group 1 and Group 2 (*p* = .67), both tested dentifrices had significantly higher % Mineral Gain than the positive control (Group 3). Micro‐CT images of representative specimens from each group are illustrated in Figure 1a−d, demonstrating the SE, DE lesion, and TE lesion.

**Table 1:** Comparison of mineral density of sound enamel and artificial enamel caries lesion between groups.

Surface	Group	Mineral density (g/cm^3^)	*p* Value
		Mean (SD)	
Sound enamel	1 SRP	2.93 (0.09)	.414^a^
	2 CTC	2.92 (0.12)	
	3 CCP	2.87 (0.14)	
	4 DI	2.95 (0.06)	
Artificial enamel caries lesion	1 SRP	1.95 (0.05)	.318^b^
	2 CTC	1.89 (0.08)	
	3 CCP	1.96 (0.04)	
	4 DI	1.97 (0.08)	

*Note*: Group 1: SRP, Sensodyne repair and protect®; Group 2: CTC, Clinpro tooth crème®; Group 3: CCP, Colgate® Cavity Protection; Group 4: DI, Distilled water.

^a^ There was no significant difference in the mean MD of sound enamel between all groups. *F* (3, 28) = 0.985, η^2^_p_ = 0.095.

^b^ There was no significant difference in the mean MD of artificial enamel caries lesion between all groups. *F* (3, 28) = 1.227, η^2^_p_ = 0.116.

**Table 2:** Comparison of mineral density of treated artificial enamel caries lesion.

Group	Mineral density (g/cm^3^)	Mineral gain (g/cm^3^)	% Mineral gain
	Mean (SD)	Mean (SD)	Mean (SD)
1 SRP	2.34 (0.06)	0.38 (0.07)	38.45 (5.48)^a^
2 CTC	2.26 (0.08)	0.36 (0.04)	35.59 (3.90)^a^
3 CCP	2.16 (0.07)	0.20 (0.04)	22.10 (4.85)^b^
4 DI	1.99 (0.08)	0.02 (0.01)	1.97 (0.64)^c^

*Note*: Group 1: SRP, Sensodyne repair and protect®; Group 2: CTC, Clinpro tooth crème®; Group 3: CCP, Colgate® Cavity Protection; Group 4: DI, Distilled water. ^a^Values with similar superscript are not significantly different (*p* = .67). ^b,c^Significantly different to other values (*p* < .01). *F* (3, 28) = 120.75, *p* < .01, η^2^ *p* = 0.93.

### 3.2 Nanoindentation mechanical characterization

In SE, EM values ranged from 103.66 (±3.04) GPa to 108.46 (±2.80) GPa in the outer region and from 104.03 (±13.17) GPa to 109.92 (±10.87) GPa in the inner enamel (Table 3). There were no significant differences in EM mean values among groups in outer (*p* = .89) and inner enamel (*p* = .78). EM values were considerably lower in DE (ICLs), ranging from 2.12 (±0.28) GPa to 4.27 (±0.87) GPa in the outer enamel and from 2.26 (±1.19) GPa to 3.31 (±1.13) GPa in the inner enamel (Table 3). Again, there were no significant differences in EM mean values among groups in the outer (*p* = .10) and inner enamel (*p* = .76).

**Table 3:** Comparison of elastic modulus of sound enamel and artificial enamel caries lesion between groups.

Surface	Group	Elastic modulus (GPa)	*p* Value
		Mean (SD)	
Sound enamel	1 SRP	104.65 (12.36)	.897^a^
(Outer layer)	2 CTC	103.66 (3.04)	
	3 CCP	108.46 (2.80)	
	4 DI	105.33 (9.74)	
Sound enamel	1 SRP	104.03 (13.17)	.783^a^
(Inner layer)	2 CTC	105.03 (2.03)	
	3 CCP	109.53 (3.03)	
	4 DI	109.92 (10.87)	
Artificial enamel caries lesion	1 SRP	3.54 (1.44)	.102^b^
(Outer layer)	2 CTC	4.27 (0.87)	
	3 CCP	2.12 (0.28)	
	4 DI	2.37 (1.13)	
Artificial enamel caries lesion	1 SRP	2.26 (1.19)	.768^b^
(Inner layer)	2 CTC	3.03 (0.86)	
	3 CCP	2.27 (0.29)	
	4 DI	3.31 (1.13)	

*Note*: Group 1: SRP, Sensodyne repair and protect®; Group 2: CTC, Clinpro tooth crème®; Group 3: CCP, Colgate® Cavity Protection; Group 4: DI, Distilled water.

^a^ There was no significant difference in the mean elastic modulus of sound enamel between all groups. Outer layer: *F* (3, 8) = 0.195, η^2^_p_ = 0.068. Inner layer: *F* (3, 8) = 0.361, η^2^_p_ = 0.119.

^b^ There was no significant difference in the mean elastic modulus of artificial enamel caries lesion between all groups. Outer layer: *F* (3, 8) = 2.894, η^2^_p_ = 0.520. Inner layer: *F* (3, 8) = 0.383, η^2^_p_ = 0.126.

In SE, H values were also statistically similar among groups in the outer enamel (*p* = .23), ranging from 4.74 (±0.42) GPa to 5.79 (±0.49) GPa, and 4.73 (±0.36) GPa to 5.70 (±0.24) GPa in the inner enamel. Again, differences among groups in inner enamel were not statistically significant (*p* = .26) (Table 4). H values in the ICL areas were also considerably lower; ranging from 0.04 (±0.03) GPa to 0.16 (±0.03) GPa in the outer layer and 0.03 (±0.02) GPa to 0.09 (±0.07) in the inner layer. There were no significant differences in H values among groups in outer (*p* = .09) and inner enamel (*p* = .26)—see Table 4.

**Table 4:** Comparison of hardness of sound enamel and artificial enamel caries lesion between groups.

Surface	Group	Hardness (GPa)	*p* Value
		Mean (SD)	
Sound enamel	1 SRP	5.59 (0.34)	.234^a^
(Outer layer)	2 CTC	4.74 (0.42)	
	3 CCP	5.79 (0.49)	
	4 DI	5.77 (0.28)	
Sound enamel	1 SRP	5.50 (0.43)	.260^a^
(Inner layer)	2 CTC	4.73 (0.36)	
	3 CCP	5.61 (0.74)	
	4 DI	5.70 (0.24)	
Artificial enamel caries lesion	1 SRP	0.09 (0.06)	.093^b^
(Outer layer)	2 CTC	0.16 (0.03)	
	3 CCP	0.05 (0.01)	
	4 DI	0.04 (0.03)	
Artificial enamel caries lesion	1 SRP	0.04 (0.02)	.266^b^
(Inner layer)	2 CTC	0.09 (0.07)	
	3 CCP	0.03 (0.02)	
	4 DI	0.06 (0.03)	

*Note*: Group 1: SRP, Sensodyne repair and protect®; Group 2: CTC, Clinpro tooth crème®; Group 3: CCP, Colgate® Cavity Protection; Group 4: DI, Distilled water.

^a^ There was no significant difference in the mean hardness of sound enamel between all groups. Outer layer: *F* (3, 8) = 1.750, η^2^_p_ = 0.396. Inner layer: *F* (3, 8) = 1.619, η^2^_p_ = 0.378.

^b^ There was no significant difference in the mean hardness of artificial enamel caries lesion between all groups. Outer layer: *F* (3, 8) = 3.035, η^2^_p_ = 0.532. Inner layer: *F* (3, 8) = 1.593, η^2^_p_ = 0.374.

When considering EM and H values of TE, both the independent factors (treatment and layer of the enamel) and the interaction of the factors (treatment*layer) were significant (*p* < .05). The dentifrice test group with f‐TCP (Group 2) had significantly higher EM values than the positive control (fluoridated dentifrice, Group 3) and the negative control (DI; Group 4) in the outer enamel layer (*p* < .05) (Table 5). When considering the inner layer, calcium sodium phosphosilicate (Group 1) and f‐TCP (Group 2) had significantly higher EM values than both the positive and negative controls (*p* < .05) (Table 5). H values in both the outer and inner enamel were significantly higher for specimens treated with dentifrices containing calcium sodium phosphosilicate (Group 1) and f‐TCP (Group 2) than the specimens from both the positive control (fluoridated dentifrice, Group 3) and the negative control (*p* < .05) (Table 6).

**Table 5:** Elastic modulus of treated artificial enamel caries lesions at 30μm (outer layer) and 90μm (inner layer) depth from surface by groups.

Variable	Group	Outer Layer	Inner Layer
		Mean (SD)	Mean (SD)
Elastic Modulus (GPa)	1 SRP	32.21 (6.69)^a,b^	20.82 (3.72)^a^
	2 CTC	35.81 (8.83)^a^	26.04 (9.87)^a^
	3 CCP	25.65 (2.94)^b^	9.67 (0.28)^b^
	4 DI	3.78 (1.82)^c^	7.18 (3.26)^b^

*Note*: Group 1: SRP, Sensodyne repair and protect®; Group 2, CTC: Clinpro tooth crème®; Group 3: CCP, Colgate® Cavity Protection; Group 4: DI, Distilled water. Values with similar superscript (a,b) in the column are not significantly different (*p* > .05).

**Table 6:** Hardness of treated artificial enamel caries lesions at 30 μm (outer layer) and 90 μm (inner layer) depth from surface by groups.

Variable	Group	Outer Layer	Inner Layer
		Mean (SD)	Mean (SD)
Hardness (GPa)	1 SRP	1.29 (0.18)^a^	0.75 (0.07)^a^
	2 CTC	1.41 (0.31)^a^	0.58 (0.21)^a^
	3 CCP	1.02 (0.19)^b^	0.39 (0.10)^b^
	4 DI	0.11 (0.08)^c^	0.07 (0.02)^c^

*Note*: Group 1: SRP, Sensodyne repair and protect®; Group 2: CTC, Clinpro tooth crème®; Group 3: CCP, Colgate® Cavity Protection; Group 4: DI, Distilled water. Values with similar superscript (a,b,c) in the column are not significantly different (*p* > .05).

## 4 DISCUSSION

The MD and nanomechanical properties of DE lesions increased by treatment with fluoridated dentifrices containing added active ingredients (Novamin® or f‐TCP) compared to treatment with standard fluoride dentifrices. The added active ingredients remineralized the carious lesions better than the standard fluoridated dentifrice and the two null hypotheses were therefore rejected.

The benefits of fluoridated dentifrices are well established and supported by reliable scientific evidence (Marinho et al., 2003; Weyant et al. 2013; Walsh et al., 2019). Additional active ingredients have been developed to supplement the beneficial effects of fluoride in such dentifrices. Other components include calcium sodium phosphosilicate and f‐TCP. Calcium sodium phosphosilicate has been added to dentifrices to reduce dental sensitivity. It leads to the formation of a carbonate‐enriched HCA layer, which is similar to tooth mineral in structure and chemical composition, leading to the remineralization of ICLs (Burwell et al., 2009). Research has shown that dentifrices containing Calcium sodium phosphosilicate (Novamin®) and fluoride are more effective than fluoride alone in enhancing remineralization and inhibiting demineralization of carious lesions (Abbasoglu et al., 2019; Manchery et al., 2019; Wang et al., 2016). However, these past studies tested the efficacy of dentifrices by analyzing the surface roughness and hardness of lesions. These tests may not accurately depict what is happening throughout the entire lesion depth, a strength of the present study. Previous studies on calcium sodium phosphosilicate‐containing dentifrices have mainly focused on its efficacy in reducing dentinal hypersensitivity (Hungund et al., 2012; Wefel, 2009).

f‐TCP prevents demineralization and increases remineralization by forming acid‐resistant minerals within the tooth matrix (Karlinsey, Mackey, Stookey, et al., 2009; Karlinsey et al., 2010). This is achieved by maintaining the stability of individual ions in dentifrices during storage, only reacting with tooth minerals upon contact with the saliva. However, few studies have shown f‐TCP as more effective in remineralizing DE lesions than fluoride alone (Buckshey et al., 2019; Rao et al., 2017; Vanichvatana & Auychai, 2013).

To replicate intraoral conditions and mineral level changes in a controlled in vitro setting, pH cycling has been employed in previous studies (Buzalaf et al., 2010; Cheng et al., 2015). It provides rapid, inexpensive tests that mimic changes in mineral levels with a high level of control. Variables can be easily controlled while delivering high levels of sensitivity. Furthermore, a smaller sample size is required for statistically significant outcomes. However, there are limitations to the pH cycling model as it cannot solely replicate complex intraoral conditions, including plaque formation, the dynamic nature and range of pH levels, and the dilution or clearance of dentifrices applied intraorally. Also, it cannot replicate the differences in periods of demineralization and remineralization intraorally (Buzalaf et al., 2010; Cheng et al., 2015). Despite this, pH cycling remains an appropriate model to test remineralization and demineralization changes in enamel.

The MD, EM, and H values of sound enamel in this investigation were similar to those reported in previous studies with similar assessment methods (Hayashi‐Sakai et al., 2019; Willems et al., 1993). The MD of sound enamel in this study averaged 2.92 g/cm^3^. A study investigating human teeth by Hayashi‐Sakai et al. found the MD of sound enamel to range from 1.39 to 4.43 g/cm^3^, with a mean value of 3.18 g/cm^3^ (Hayashi‐Sakai et al., 2019). This study's mean EM and H of sound enamel were 106.32 and 5.42 GPa, respectively, which was comparable to values previously reported (EM: 90.58 ± 16.13 GPa, H 3.39 ± 1.80 GPa) (Willems et al., 1993). The results obtained via Micro‐CT and nanomechanical testing showed that dentifrices with added calcium sodium phosphosilicate and f‐TCP were more effective in remineralizing ICLs than fluoridated toothpaste alone.

The nanoindentation testing showed a significant difference in EM and H in the outer layers at 30 µm from the enamel surface in the dentifrice test group with f‐TCP (Group 2), compared to the positive control (fluoridated dentifrice, Group 3). In the inner layer at 90 µm from the surface, Groups 1 and 2 performed better than Group 3. Adding f‐TCP seems to be effective in remineralizing both the surface and the inner layers of DE lesions, and it seems to allow deeper penetration into the lesion, improving its efficacy in enamel remineralization. This may mean that while dentifrices containing calcium sodium phosphosilicate or f‐TCP were more effective in remineralization across the whole depth of the lesion, fluoride‐only dentifrices may be as effective in preventing surface ICLs from progressing into cavities.

While previous studies on the remineralizing potential of Calcium sodium phosphosilicate and f‐TCP on DE lesions investigated the MD or surface hardness of the treated lesions, the results are comparable to those obtained in this study. Balakrishnan et al. found an MD percentage increase of 33.9% and 34.5% in ICLs treated with Sensodyne repair and protect® and Clinpro tooth crème®, respectively (Balakrishnan et al., 2013). This finding aligns with our present study's results, where the MD percentage increase was 38.4% and 35.5%, respectively. In addition, Zerrin et al. found a 20.6% increase in surface hardness of lesions treated with Sensodyne repair and protect® (Abbasoglu et al., 2019), while Rao et al. and Lippert et al. found a 25% and 54.8% increase in surface hardness of lesions treated with Clinpro tooth crème®, respectively (Lippert & Gill, 2019; Rao et al., 2017). As these other studies only investigated H on the surface of lesions, while this study investigated EM and H across the depth of ICLs, the results are not fully comparable, although they were similar.

As outlined above, this study had some limitations. Despite being widely accepted, pH cycling models cannot truly replicate the complex intraoral environment, including fluctuations in pH and buffering salivary action. Thus, the results reported here might not be the same as in in vivo conditions. Our study also relied on sound extracted teeth from different patients. Although no anomalies or pathology were visible, it is possible that differences in enamel structure and varying exposure to fluoride during life exist between specimens analyzed. However, similar average values in MD and mechanical properties in sound enamel among test groups suggest these differences would have been negligible.

Although caries is a common disease, the difficulty of reducing risk factors and lifestyle choices makes it a complex condition to control or manage at an individual and community level (Banoczy, 2013). Using effective dentifrices would benefit populations with limited access to dental care and decrease the incidence of cavitated carious lesions. Subsequently, this could reduce the burden of treatment costs to patients and funding bodies. Future studies should investigate the elemental composition of enamel remineralized by these added active ingredients and its resistance to further acid challenges.

## 5 CONCLUSIONS

While all tested dentifrices effectively remineralized ICLs, the MD, EM, and hardness of ICLs significantly increased with the addition of Calcium sodium phosphosilicate or f‐TCP to fluoridated dentifrices compared to standard fluoride dentifrices. The added active ingredients penetrated and remineralized the deeper parts of the ICLs better than the standard fluoridated dentifrices with no such added active ingredients.

## AUTHOR CONTRIBUTIONS

*Conceptualization*: Manikandan Ekambaram. *Methodology*: Manikandan Ekambaram, Carolina Loch, Shafiq Aziz, Kai Chun Li, Robert Anthonappa, and Alison Meldrum. *Investigation*: Shafiq Aziz. *Resources*: Manikandan Ekambaram, Carolina Loch, and Kai Chun Li. *Data curation*: Manikandan Ekambaram, Carolina Loch, Shafiq Aziz, Kai Chun Li. *Writing‐original draft preparation*: Shafiq Aziz. *Writing—review and editing*: Manikandan Ekambaram, Carolina Loch, Shafiq Aziz, Kai Chun Li, Robert Anthonappa, and Alison Meldrum. *Supervision*: Manikandan Ekambaram, Carolina Loch, and Alison Meldrum. *Project administration*: Manikandan Ekambaram and Carolina Loch. *Funding acquisition*: Manikandan Ekambaram, Carolina Loch, Robert Anthonappa, and Alison Meldrum.

## CONFLICT OF INTEREST STATEMENT

The author declare no conflict of interest.

## Data Availability

The data that support the findings of this study are available from the corresponding author upon reasonable request.
